# Surgery shows survival benefit in patients with primary intestinal diffuse large B‐cell lymphoma: A population‐based study

**DOI:** 10.1002/cam4.3882

**Published:** 2021-05-01

**Authors:** Moran Wang, Shengling Ma, Wei Shi, Yuanyuan Zhang, Shanshan Luo, Yu Hu

**Affiliations:** ^1^ Institute of Hematology Union Hospital Tongji Medical College Huazhong University of Science and Technology Wuhan China; ^2^ Department of Oncology Tongji Hospital Tongji Medical College Huazhong University of Science and Technology Wuhan China

**Keywords:** nomogram, primary intestinal diffuse large B‐cell lymphoma, prognosis, SEER, surgery

## Abstract

**Background:**

The clinical characteristics and prognosis of primary intestinal diffuse large B‐cell lymphoma (PI‐DLBCL) are rarely reported. We aimed to explore the role of surgery in patients with PI‐DLBCL.

**Methods:**

Adult PI‐DLBCL patients were included from the Surveillance, Epidemiology, and End Results database. The effect of surgery was evaluated by Kaplan–Meier and Cox proportional regression analyses. Propensity score matching (PSM) was used to reinforce our results. Lasso regression was utilized to determine independent risk factors of overall survival (OS) for a nomogram and a novel web‐based calculator. The performance of the model was measured via concordance index, receiver operating characteristic curve, and calibration plots in both cohorts.

**Results:**

Overall, 1602 patients with PI‐DLBCL were analyzed. Surgery significantly improved survival in both univariate and multivariate analyses (*p* = 0.007, *p* < 0.001, respectively). Before PSM, local tumor destruction (LTD) displayed a survival advantage over resection in patients without chemotherapy (*p* = 0.034). After PSM, surgery was still identified as a beneficial factor for OS (*p* = 0.0015). However, there was no statistical difference between LTD and resection (*p* = 0.32). The nomogram for 3‐, 5‐, and 10‐year OS predictions exhibited dependable consistency between internal and external validation.

**Conclusion:**

This study approves the beneficial effect of surgery on clinical endpoints in PI‐DLBCL patients. For those who are not suitable for resection, LTD may also be a practical option. The predictive nomogram and the web‐based calculator could help clinicians individually evaluate the prognosis and optimize personalized treatment decisions for these patients.

## Graphical abstract

This study approves the beneficial effect of surgery on clinical outcome in patients with primary intestinal diffuse large B‐cell lymphoma. The predictive nomogram and the web‐based calculator could help clinicians to predict the prognosis and optimize individualized treatment decisions for these patients.

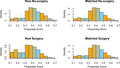

## 1 INTRODUCTION

The gastrointestinal (GI) tract is one of the most typical sites of primary extranodal non‐Hodgkin's lymphoma, accounting for 30%–45% of all sites.^1^, ^2^ Gastric lymphoma ranks first (55%–70%), followed by small intestinal lymphoma (20%–35%) and colorectal lymphoma (5%–10%).^3^, ^4^ Among different primary intestinal lymphoma (PIL) types, diffuse large B‐cell lymphoma (DLBCL) is the most frequent one as indicated by several studies.^5^, ^6^, ^7^, ^8^ Compared with gastric lymphoma, the current cognition of PIL is limited, because PIL is generally investigated as a subset of GI lymphomas.^9^, ^10^ Several studies have shown that the predictors of gastric and intestinal DLBCLs are different.^11^, ^12^, ^13^, ^14^ Patients with PI‐DLBCL have a lower overall survival (OS) rate and a joint of chemotherapy and surgery is usually required because of the increased incidence of complications.^15^, ^16^

Although some prognostic factors related to PI‐DLBCL have been proposed, including patient status, clinical stage, biochemical anomalies, and histological subtypes, the relevance of these indicators to optimal remedy remains unclear.^4^, ^7^, ^16^, ^17^ Chemotherapy is an essential option for the management of DLBCL, and the utilization of anti‐CD20 antibody rituximab has improved the survival rate of most DLBCL patients in the past two decades.^18^ However, surgical resection is only recommended under specific circumstances. Results of GI lymphoma studies are debatable regarding the benefit of surgical excision.^3^, ^19^ In recent years, many researchers recommended a combined therapy of chemotherapy and surgery to improve OS.^13^, ^20^, ^21^ Due to the potential risk of death and decreased quality of life, the contribution of surgery to PI‐DLBCL needs to be reassessed. Whereas a large clinical trial is unrealistic to carry out due to the scarcity and heterogeneity of PI‐DLBCL.

In this study, we utilized population‐based data from the Surveillance, Epidemiology, and End Results (SEER) database to explore the relationship between surgery and the clinical outcome of PI‐DLBCL patients to clarify its value. Besides, we established a practical web‐based calculator for individual survival evaluation of patients with PI‐DLBCL.

## 2 METHODS

### 2.1 Data and cohort definition

We extracted patients diagnosed with PI‐DLBCL from 2004 to 2016 in the SEER 18 registries. PI‐DLBCL was identified according to the histology code (9680) and primary anatomic site (C17‐21.8) of the International Classification of Diseases for Oncology Third Edition (ICD‐O‐3). Patients who were microscopically confirmed with PI‐DLBCL were included. We excluded these patients: (1) under 18 years old; (2) DLBCL was not the first primary malignancy; (3) with no information on Ann Arbor stage, race, or marital status; (4) unknown surgery treatment, A symptom or B symptom; (5) survival time was recorded as zero.

### 2.2 Definition of variables

The demographics and disease characteristics of patients included age at diagnosis, sex, race, marital status, primary site, Ann Arbor stage, symptom, surgery, chemotherapy, survival time, and vital status. Age was divided into four categories (18–59, 60–69, 70–79, or ≥80 years), race into white, black, and other. Marital status was classified into married, single, and other. Stage was categorized as early (Ann Arbor Stage I/Ⅱ) and advanced stage. Primary site was divided into three sites: small intestine (C17.0–17.9), colon (C18.0–18.9), and anorectal (C19.9–21.8). Surgery group was partitioned into local tumor destruction (LTD, including photodynamic therapy/electrocautery/cryosurgery/laser/polypectomy) and resection (including partial resection/radical resection).

### 2.3 Propensity score matching

In retrospective studies, selection bias is inevitable, resulting in uneven distribution of confounding factors between two groups. To reduce selection bias and adjust for the confounding factors, we carried out a propensity score matching (PSM) for the surgery variable accounting for all the covariates mentioned above.^22^, ^23^ We chose 1:1 nearest neighbor matching with a caliper of 0.01 to accept a matched pair.^24^, ^25^ Cox proportional hazard model was used for survival analyses of the two matched groups.

### 2.4 Statistical analysis

Summary statistics were applied to depict the basic characteristics of the included population. The survival curves were plotted with Kaplan–Meier method and evaluated via log‐rank tests. To analyze independent prognostic factors related to the OS, we used both univariate and multivariate Cox regression to compute hazard ratio (HR) and the 95% confidence interval (CI).

Furthermore, a nomogram model and a web‐based application were established to predict the 3‐, 5‐, and 10‐year survival probabilities for PI‐DLBCL patients. L1‐penalized (Lasso) regression was implemented to filter factors for the OS nomogram.^26^ All patients were indiscriminately allocated to the training set and the validation set at a ratio of 3:1 as previously mentioned.^27^ The accuracy of the nomogram was verified by a bootstrapped resample with 500 iterations. The discrimination of the nomogram was assessed via concordance index (C‐index) and the area under the curve (AUC) value of the time‐dependent receiver operating characteristic curve (ROC).^28^, ^29^ Calibration plots were generated to verify the unbiased estimation of outcomes. All statistical analyses were executed using the R software version 3.6.2, SEER*stat 8.3.8 and SPSS version 25. Statistical tests were bilateral and significance was set as *p* < 0.05.

## 3 RESULTS

### 3.1 Characteristics of patients

**FIGURE 1:**
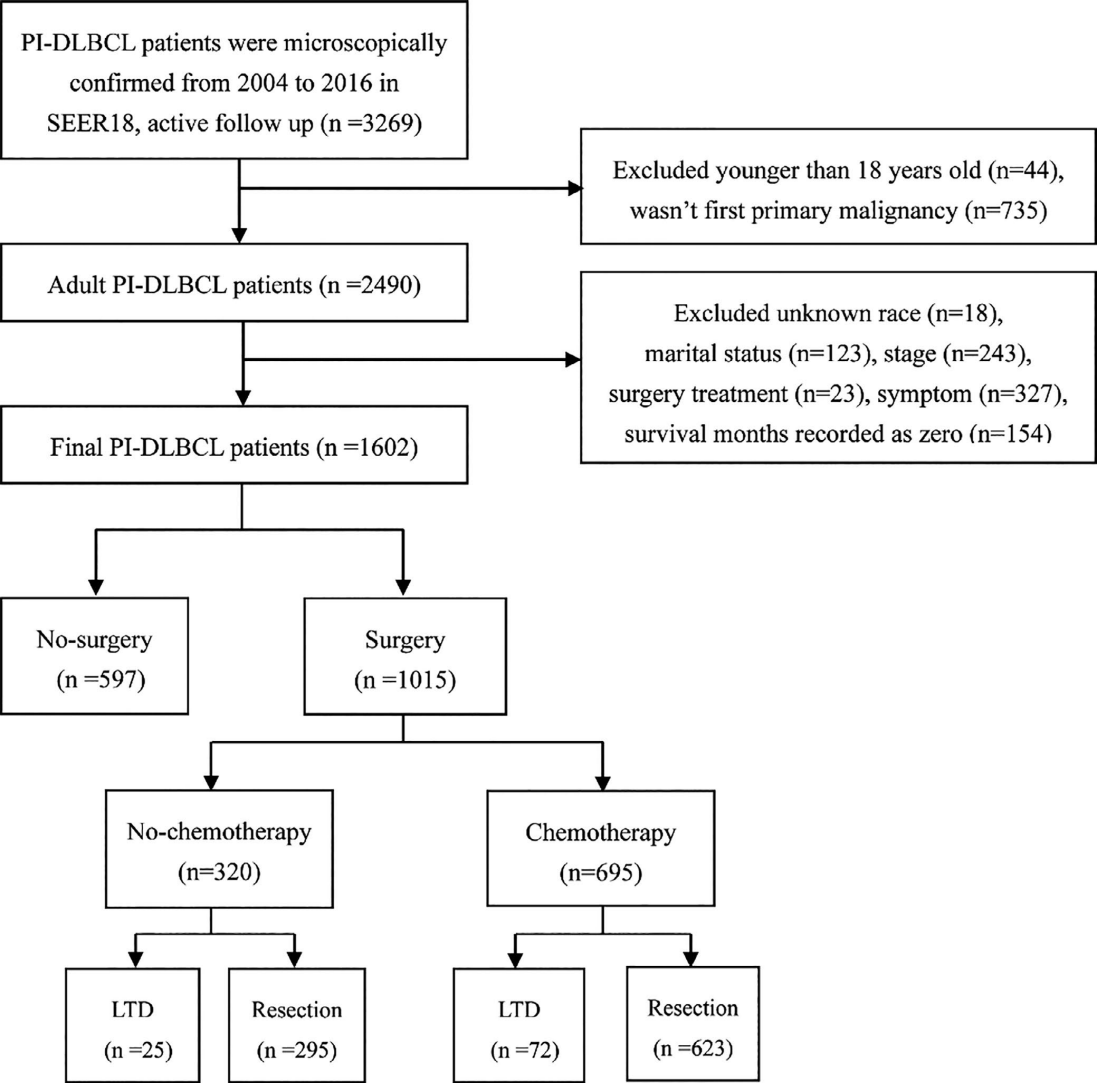
Flow chart for screening eligible patients. PI‐DLBCL, primary intestinal diffuse large B‐cell lymphoma

Upon applying the inclusion and exclusion criteria, we included 1602 adults confirmed with PI‐DLBCL in the SEER database from 2004 to 2016 (Figure 1). Demographic characteristics and treatment information of patients in the whole cohort are shown in Table 1. The median age at the time of diagnosis was 66 years (range 18–99). Most patients were male (63.3%), white (83.3%), and mainly in the early stage (70.7%). The small intestine (56.8%) was more vulnerable than colon and anorectal regions. The majority of patients underwent surgery (1015: 63.4%) and chemotherapy (1202: 75.0%). Moreover, we noted that patients without chemotherapy were more likely to receive surgery (320: 80.0%).

**TABLE 1:** Demographic characteristics of patients

Parameters	Before PSM				After PSM		
	Total	No surgery	Surgery	*p* ^a^	No surgery	Surgery	*p* ^a^
	1602	587 (36.6%)	1015 (63.4%)		515	515	
Age, years				0.064			0.793
18–59	582 (36.3%)	237 (40.4%)	345 (34.0%)		203 (39.4%)	197 (38.3%)	
60–69	355 (22.2%)	116 (19.8%)	239 (23.5%)		106 (20.6%)	117 (22.7%)	
70–79	371 (23.2%)	129 (22.0%)	242 (23.8%)		114 (22.1%)	117 (22.7%)	
≥80	294 (18.4%)	105 (17.9%)	189 (18.6%)		92 (17.9%)	84 (16.3%)	
Sex				0.309			0.648
Male	1014 (63.3%)	381 (64.9%)	633 (62.4%)		337 (65.4%)	330 (64.1%)	
Female	588 (36.7%)	206 (35.1%)	382 (37.6%)		178 (34.6%)	185 (35.9%)	
Race				0.743			0.775
White	1335 (83.3%)	485 (82.6%)	850 (83.7%)		432(83.9%)	435(84.5%)	
Black	94 (5.9%)	34 (5.8%)	60 (6.0%)		28 (5.4%)	31 (6.0%)	
Other	173 (10.8%)	68 (11.6%)	105(10.3%)		55 (10.7%)	49 (9.5%)	
Marital status				0.021			0.243
Married	929 (58.0%)	316 (53.8%)	613 (60.4%)		293 (56.9%)	296 (57.5%)	
Single	311 (19.4%)	132 (22.5%)	179 (17.6%)		117 (22.7%)	98 (19.0%)	
Other	362 (22.6%)	139 (23.7%)	223 (22.0%)		105 (20.4%)	121 (23.5%)	
Primary site				<0.001			0.786
Small intestine	910 (56.8%)	298 (50.8%)	612 (60.3%)		290 (56.3%)	291 (56.5%)	
Colon	595 (37.1%)	224 (38.2%)	371 (36.6%)		194 (37.7%)	198 (38.4%)	
Anorectal	97 (6.1%)	65 (11.1%)	32 (3.2%)		31 (6.0%)	26 (5.0%)	
Stage				0.012			0.946
I/II	1133 (70.7%)	393 (67.0%)	740 (72.9%)		361 (70.1%)	362 (70.3%)	
III/IV	469 (29.3%)	194 (33.0%)	275 (27.1%)		154 (29.9%)	153 (29.7%)	
Symptom				0.082			0.374
A	1136 (70.9%)	401 (68.3%)	735 (72.4%)		357 (69.3%)	370 (71.8%)	
B	466 (29.1%)	186 (31.7%)	280 (27.6%)		158 (30.7%)	145 (28.2%)	
Chemotherapy				<0.001			0.721
Yes	1202 (75.0%)	507 (86.4%)	695 (68.5%)		440 (85.4%)	444 (86.2%)	
No/unknown	400 (25.0%)	80 (13.6%)	320 (31.5%)		75 (14.6%)	71 (13.8%)	

Abbreviation: PSM, propensity score matching.

^a^ *p*‐value from chi‐square tests.

### 3.2 Association of surgery with overall survival

**FIGURE 2:**
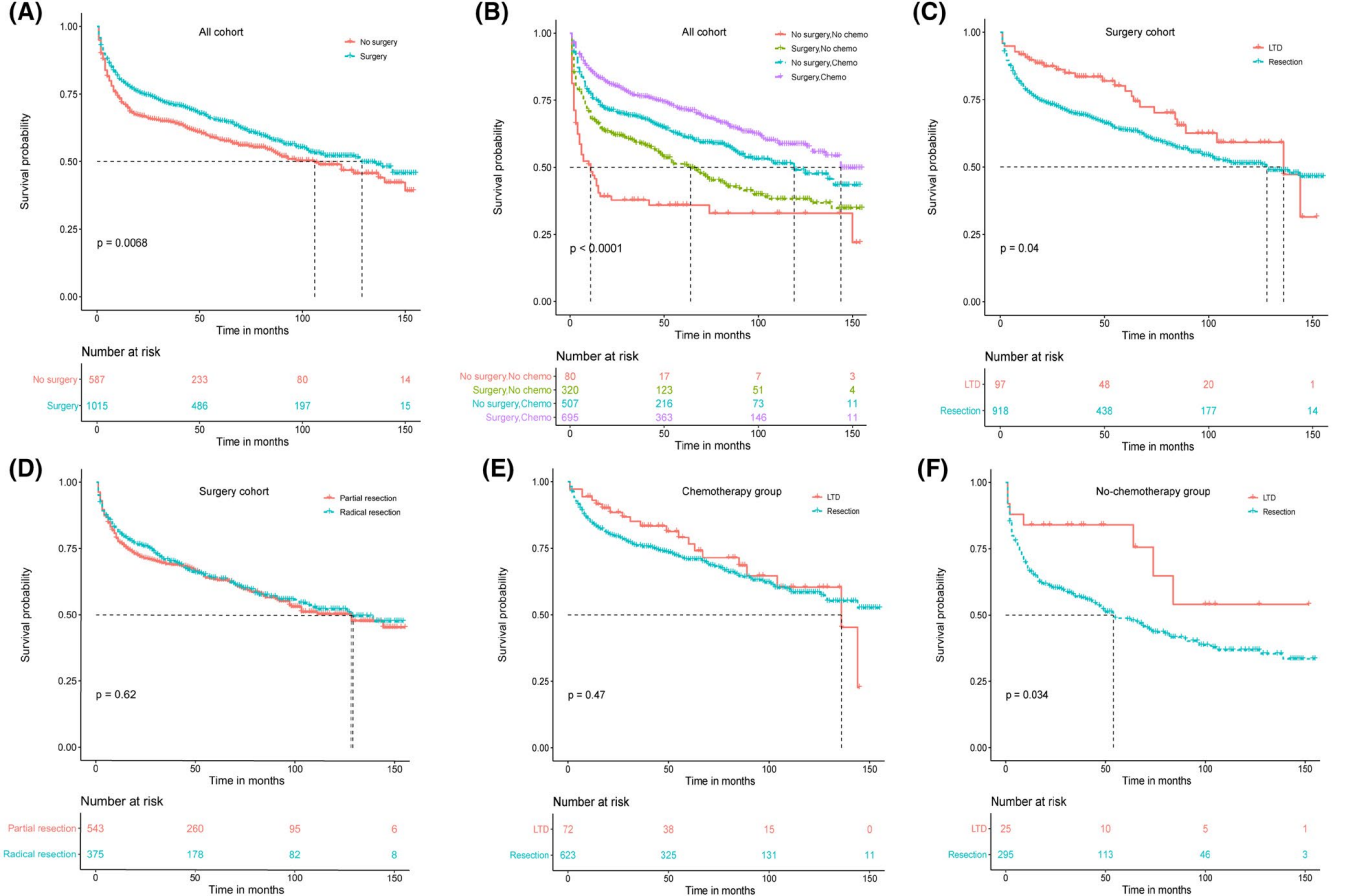
Kaplan–Meier curves of overall survival before PSM. (A) No‐surgery versus surgery in all cohorts; (B) combined effect of surgery and chemotherapy in all cohorts; (C) LTD versus resection in surgery cohorts; (D) Partial resection versus radical resection; (E) LTD versus resection in chemotherapy group; (F) LTD versus resection in no‐chemotherapy group. LTD, local tumor destruction; PSM, propensity score matching

In total, 3‐, 5‐, and 10‐year survival rates in the whole cohort were 68.7% (95% CI, 66.3–71.1), 62.4% (95% CI, 59.9–64.9), and 50.2% (95% CI, 47.1–53.3). The 3‐, 5‐, and 10‐year OS probabilities were 71.1%, 65.0%, and 52.0%, respectively, in patients undergoing surgery, and 64.5%, 57.9%, and 46.8%, respectively, for patients without surgery. Kaplan–Meier survival curves for surgery and no‐surgery groups indicated that surgery was related to better survival (*p* = 0.0068; Figure 2A). Combining surgery and chemotherapy presented optimal outcome (*p* < 0.001; Figure 2B). Univariate analysis showed that age at diagnosis, married status, stage, surgery treatment, and chemotherapy were significantly related to OS (all *p* < 0.05). Although not statistically significant (*p* = 0.093), B symptom manifested worse survival. According to the previous research, B symptom is an important factor affecting clinical outcome,^30^, ^31^, ^32^ so we also included it in the multivariate Cox analysis. On multivariate Cox analysis, age at diagnosis, stage, symptom, chemotherapy, and surgery treatment were independent prognostic indicators (Table 2).

**TABLE 2:** Prognostic factors for overall survival

	Univariate		Multivariate		Propensity score cox regression^b^	
	HR (95% CI)	*p* ^a^	HR (95% CI)	*p* ^a^	HR (95% CI)	*p* ^a^
Age, years		<0.001		<0.001		<0.001
18–59	Reference		Reference		Reference	
60–69	1.59 (1.25–2.03)		1.62 (1.27–2.07)		1.62 (1.20–2.20)	
70–79	2.39 (1.91–2.99)		2.34 (1.87–2.93)		2.36 (1.77–3.15)	
≥80	5.16 (4.16–6.41)		4.57 (3.66–5.71)		4.89 (3.69–6.50)	
Sex		0.234				0.084
Male	Reference		—		Reference	
Female	1.09 (0.94–1.29)		—		0.83 (0.68–1.02)	
Race		0.560				0.021
White	Reference				Reference	
Black	1.06 (0.77–1.46)		—		1.71 (1.12–2.60)	
Other	0.88 (0.68–1.14)		—		0.83 (0.59–1.15)	
Marital status		<0.001		0.112		0.463
Married	Reference		Reference		Reference	
Single	0.92 (0.75–1.14)		1.24 (0.99–1.55)		1.08 (0.82–1.42)	
Other	1.55 (1.30–1.85)		1.12 (0.94–1.35)		1.17 (0.91–1.51)	
Primary site		0.674				0.571
Small intestine	Reference		—		Reference	
Colon	1.06 (0.90–1.24)		—		0.95 (0.77–1.17)	
Anorectal	1.12 (0.81–1.55)		—		1.20 (0.78–1.84)	
Stage		<0.001		<0.001		<0.001
I/II	Reference		Reference		Reference	
III/IV	1.62 (1.38–1.90)		1.61 (1.37–1.90)		1.59 (1.30–1.95)	
Symptom		0.093		0.027		0.031
A	Reference		Reference		Reference	
B	1.15 (0.98–1.36)		1.21 (1.02–1.43)		1.26 (1.02–1.55)	
Chemotherapy		<0.001		<0.001		<0.001
No/unknown	Reference		Reference		Reference	
Yes	0.53 (0.45–0.63)		0.58 (0.48–0.69)		0.54 (0.42–0.69)	
Surgery		0.007		<0.001		<0.001
No surgery	Reference		Reference		Reference	
Surgery	0.81 (0.69–0.94)		0.69 (0.58–0.81)		0.68 (0.56–0.83)	

Abbreviations: CI, confidence interval; HR, hazard ratio.

^a^ *p* values are derived from Cox proportional hazard model and Log rank test.

^b^ Full model multivariable cox regression analysis after propensity score Cox regression.

### 3.3 Subgroup analyses

In order to explore the survival advantage of surgery in certain subsets of patients, we performed a stratified analysis showing that surgery achieved better survival in the male group, white and black group, married group, small intestine group, early stage group, patients with B symptom as well as elderly patients (≥70 years old) (all *p* < 0.05; Figure S1).

### 3.4 Effect of surgical mode on survival

Furthermore, we aimed to investigate the effect of surgery mode on the clinical endpoint of PI‐DLBCL. Firstly, we analyzed the entire population who underwent surgery. We found that LTD was associated with a survival benefit over resection (Figure 2C; *p* = 0.04), and there was no difference between partial resection and radical resection (Figure 2D; *p* = 0.62). Considering the possible confounding effects of chemotherapy, we divided the population into the chemotherapy group and the non‐chemotherapy group. We performed a stratified analysis of the surgery variable in both groups. We concluded that the way of surgery had no significant effect on patients receiving chemotherapy (Figure 2E; *p* = 0.47). However, for patients without chemotherapy, LTD showed better OS than resection (Figure 2F; *p* = 0.034).

### 3.5 Propensity score matching for surgical excision

**FIGURE 3:**
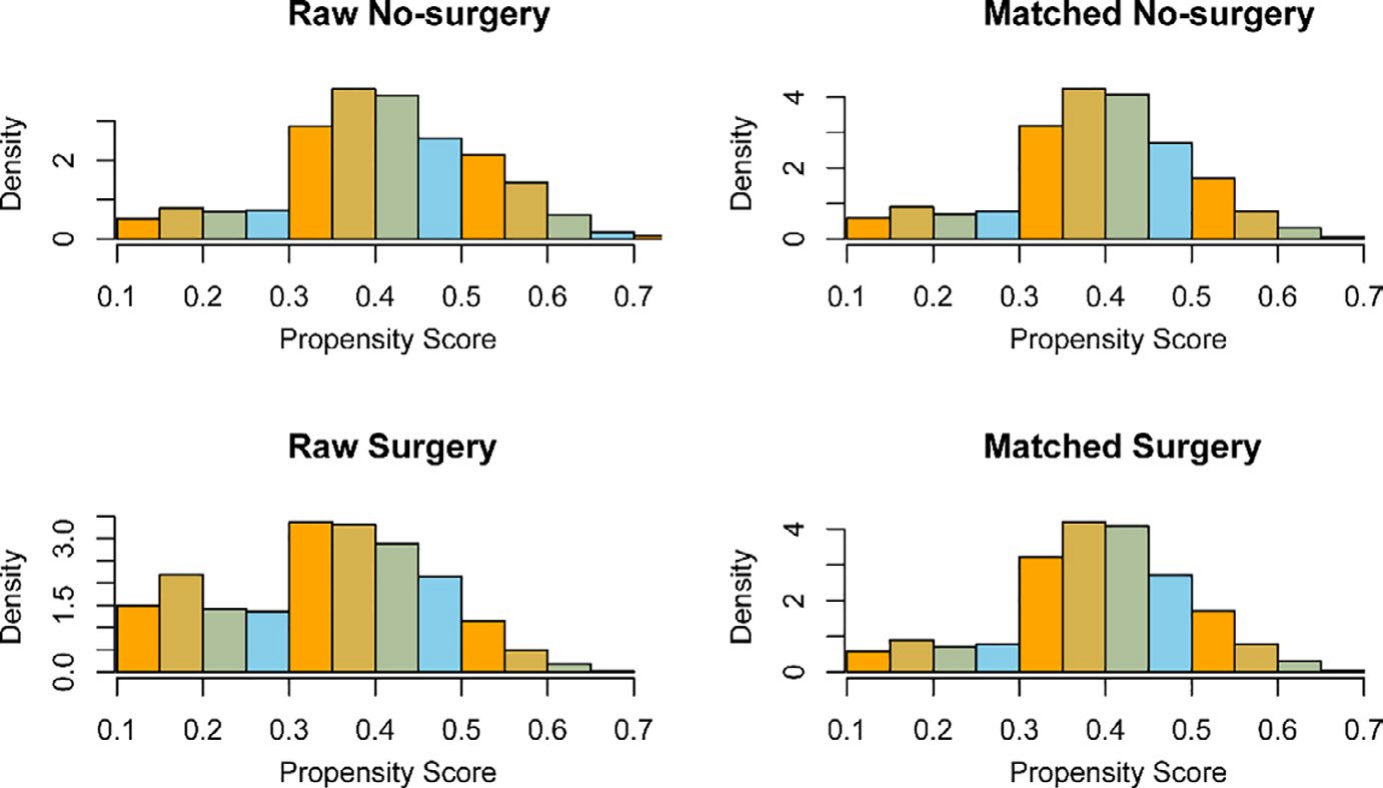
The histogram of raw data and matched data for surgery. The left side is the histogram before matching and the right side is the histogram after matching. The resemblance between the surgery and no‐surgery group was associated with the achievement of matching

**FIGURE 4:**
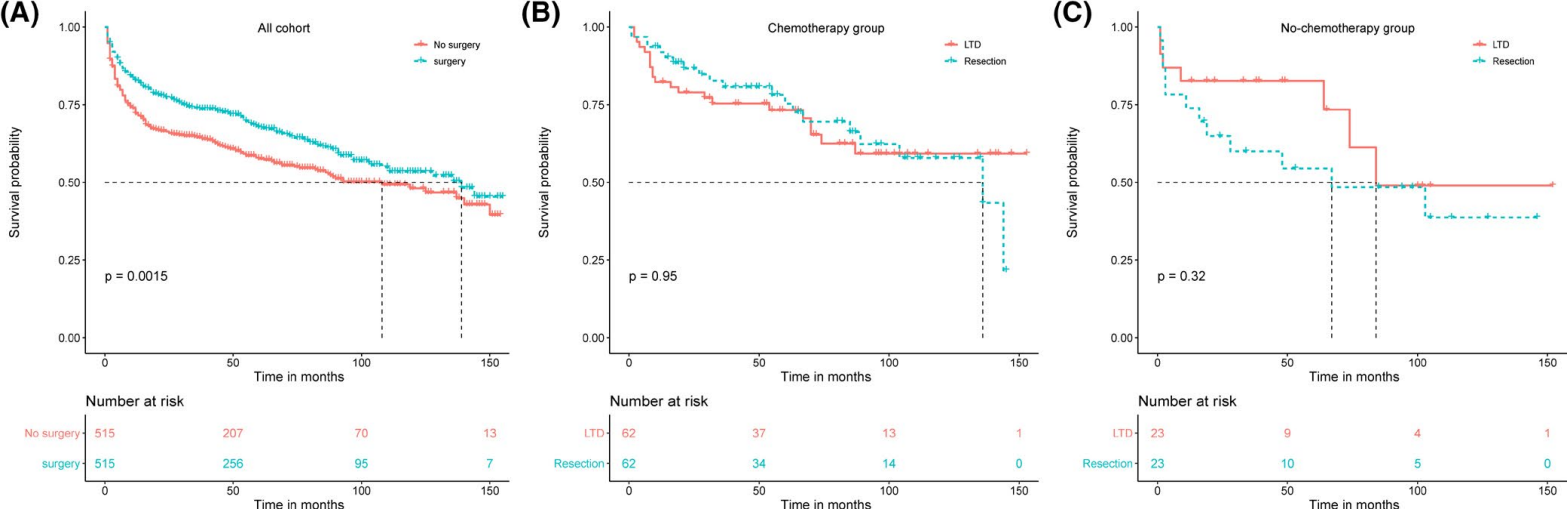
Kaplan–Meier curves of overall survival after PSM. (A) No surgery versus surgery in all cohorts. (B) LTD versus resection in chemotherapy group. (C) LTD versus resection in no‐chemotherapy group. LTD, local tumor destruction; PSM, propensity score matching

PSM was introduced to optimize the imbalance among the aforementioned baseline variables in all cohorts. As shown in Figure 3, the similarity of histograms after PSM (right side ones) was significantly higher than those without PSM on the left side, indicating that potential selection bias related to surgical treatment was minimized. After PSM, surgical treatment still had a survival advantage (HR = 0.73, 95% CI 0.60–0.89, *p* = 0.0015). The Kaplan–Meier survival curve of the new matching data was depicted in Figure 4A. We also performed PSM on surgical population of the chemotherapy group and non‐chemotherapy group, respectively (Tables S1 and S2). After PSM, we found that surgical mode did not affect the OS of both chemotherapy and non‐chemotherapy groups (Figure 4B,C).

### 3.6 Nomogram construction and internal validation

**FIGURE 5:**
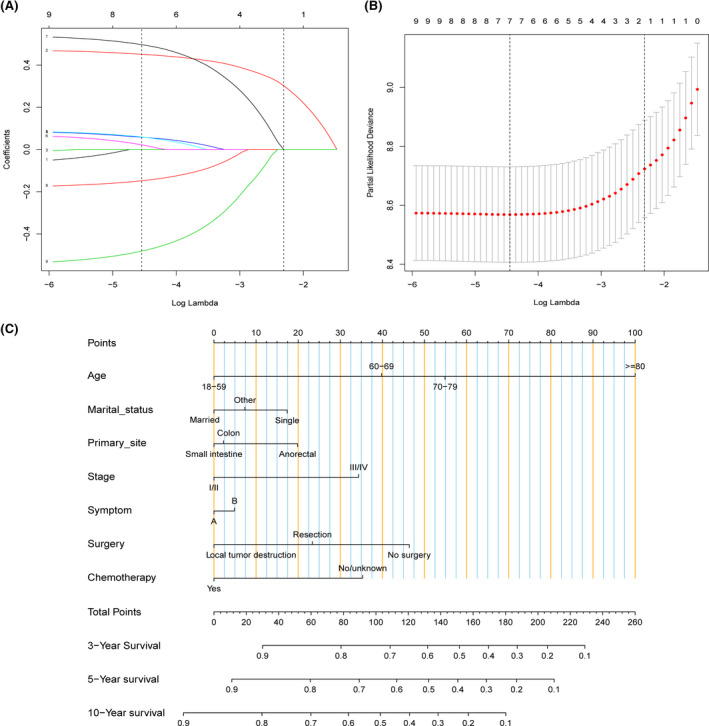
(A) LASSO coefficient distribution of predictive factors. The vertical lines drawn on the left and right represent the optimal values determined by the minimum criterion and 1‐SE criterion, respectively. Seven variables were identified (age, marital status, primary site, stage, surgery, symptom, and chemotherapy) for overall survival. (B) Identification of the optimal lambda. Ten‐fold cross‐validation and minimum criterion were used to adjust the penalty coefficient *λ* in lasso model. An optimal *λ* 0.012, with log(*λ*) = −4.45, was selected. (C) Development of the nomogram for evaluating the probability of 3‐, 5‐, and 10‐year overall survival

**FIGURE 6:**
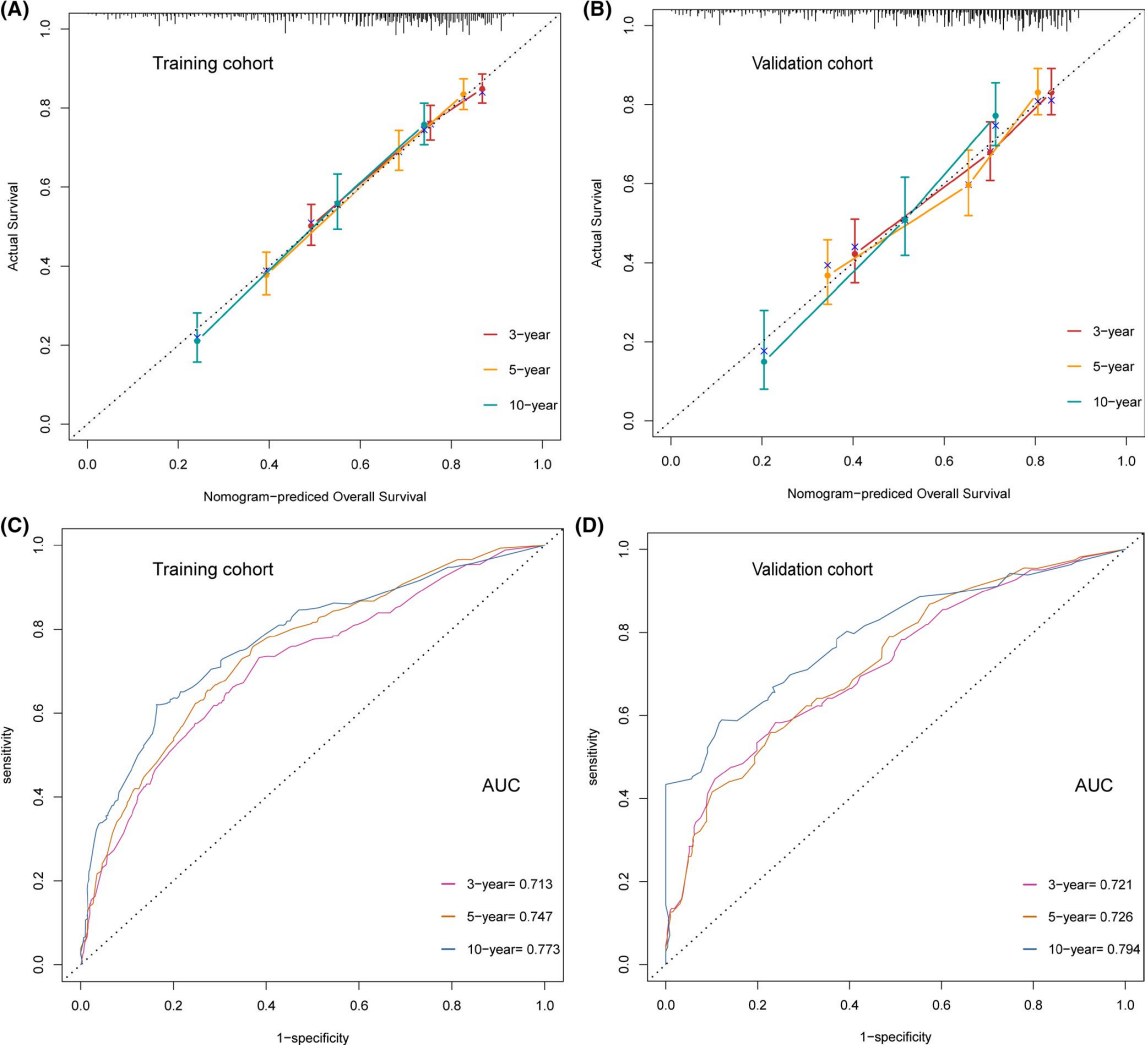
Calibration curves of the nomogram to predict the overall survival rate at 3‐, 5‐, and 10‐year in (A) training cohort; (B) validation cohort. The receiver operating characteristic curve analysis for predicting 3‐, 5‐, and 10‐year overall survival in (C) training cohort; (D) validation cohort. AUC, the area under the curve

Furthermore, a nomogram was produced to predict 3‐, 5‐, and 10‐year OS for PI‐DLBCL patients. 1122 patients were randomly divided into the training set and 480 patients to the validation set. We utilized Lasso regression model in the training cohort to identify independent risk factors affecting OS (Figure 5A,B). A total of seven prognostic factors (age, marital status, primary site, stage, surgery, symptom, and chemotherapy) were included in the nomogram for OS (Figure 5C). The C‐indexes for OS in internal and external validations were 0.703 and 0.694, respectively. Calibration curves displayed high consistency between the nomogram‐predicted survival and the actual outcome in the training cohort (Figure 6A) and the validation cohort (Figure 6B). The time‐dependent ROC analysis also exhibited good predictive accuracy of the nomogram model for OS in training cohort (3‐year AUC, 0.713; 5‐year AUC, 0.747; 10‐year AUC, 0.773; Figure 6C) and validation cohort (3‐year AUC, 0.721; 5‐year AUC, 0.726; 10‐year AUC, 0.794; Figure 6D).

### 3.7 Development of a web‐based calculator

Finally, we established a dynamic web‐based calculator accessible via https://tumor.shinyapps.io/PI‐DLBCL/ to predict the OS of patients with PI‐DLBCL based on our nomogram (Figure S2). It is convenient to predict survival probability and its 95% CI by inputting their clinical features. For example, for a married 65‐year‐old patient with stage II PI‐DLBCL, presenting B symptom, after receiving resection and chemotherapy, the 5‐year OS rate was approximately 77.0% (95% CI, 72.0–84.0).

## 4 DISCUSSION

PI‐DLBCL is a rare and heterogeneous disease entity. The lack of prospective randomized clinical trials results in the undetermined optimal therapeutic strategy. Treatments of PI‐DLBCL vary from chemotherapy alone to multimodality combined with surgery and radiotherapy. As surgical excision is not better than chemotherapy combined with radiotherapy, the role of surgery has been weakened in primary gastric DLBCL, and treatment attention has shifted to organ preservation.^33^, ^34^ However, many studies have suggested that gastric lymphoma and intestinal lymphoma yielded different survival rates and prognostic factors.^3^, ^9^, ^19^, ^35^ From the perspective of histologic lymphoma subtypes, gastric lymphoma usually presents as the mucosa‐associated lymphoid tissue subtype, with a 75% response rate to *Helicobacter pylori* eradication alone.^36^ While intestinal lymphoma is mainly dominated by the DLBCL subtype, which is more aggressive and prone to complications such as bleeding, perforation, and stenosis, contributing to the combination of surgery and chemotherapy for treatment.^16^, ^17^, ^37^, ^38^ The benefits of surgery can be attributed to some biological reasons. Firstly, intestinal lymphoma is more likely to involve the ileocecum, a site that is difficult to reach by routine endoscopy, making diagnosis difficult.^4^ Surgery can obtain biopsy tissue for early pathological diagnosis and clinical staging, laying the foundation for subsequent treatment.^39^ Secondly, surgery can remove the primary tumor lesions and some lymph nodes that may metastasize, alleviate the burden of chemotherapy and radiotherapy, and relieve tumor‐related acute complications.^40^, ^41^ Several retrospective studies have found that surgery contributes to a better outcome in PI‐DLBCL patients. Kako et al. reported that surgery before other treatments favorably led to failure‐free survival, with encouraging results for all patients who had undergone complete resection of small intestinal lesions.^42^ Hong et al. conducted an analysis involving 82 patients to explore the effect of surgery in small intestinal lymphoma, which showed that gross resection contributed to the enhancement of progression‐free survival without obviously increasing the risk of complications.^21^ A recent study showed that surgery before chemotherapy is an effective and secure treatment for small intestinal NHL, as it can prevent chemotherapy‐related perforation.^20^, ^43^

In our study, 63.4% of patients underwent surgery and 43.4% received surgery combined with chemotherapy, the multimodal treatment related to a better outcome than chemotherapy alone. As the correlation between variables tends to cause confounding bias in measuring baseline variables, PSM is often utilized to eliminate bias in observational studies.^44^ Before PSM, results showed that surgery led to OS benefits, with LTD showing superior survival over resection in patients without chemotherapy. But the difference between LTD and resection disappeared after PSM. Our finding supports the beneficial role of surgery (LTD or resection) in PI‐DLBCL patients, which is consistent with the previous report by Zhao et al. that surgery is a protective factor for prognosis regardless of whether the surgical mode is radical resection or palliative procedures.^45^

Considering the heterogeneity of PI‐DLBCL, we verified the survival benefit of surgery in critical clinical subgroups. Stage and age were prognostic factors as indicated in many reports.^3^, ^21^, ^46^ Patients with stage I/Ⅱ could benefit from surgery in our study. Still, this advantage did not exist in advanced patients, in accordance with previous results.^47^ Given the high risk of complications and death associated with surgery in elderly patients, it is essential to assess the contribution of surgery to them. Our results also verified the benefits among these patients. The small intestine is the most typical location of PI‐DLBCL in this study, which is accordant with previous studies.^7^, ^8^, ^17^ Small intestine DLBCL lymphoma is often along with initial obstruction or perforation, so surgery is usually a mandatory first‐line treatment giving a favorable prognosis (5‐year OS: 82%).^48^ Kaplan–Meier survival analysis showed that surgery yielded better survival in the small intestine group.

In multiple regression analysis, the multicollinearity between variables will affect the research conclusions. Lasso Cox regression analysis was introduced to screen variables during the nomogram construction in our study to cope with potential collinearity. In the Lasso regression model, variables are assigned to different penalties. The more important variables are punished less, making them more likely to be retained in the model, while the less critical variables are punished more and tend to be discarded. Therefore, this method can select the most important prognostic factors to build a model to predict survival.^26^, ^49^

This study presents several limitations. Firstly, although PSM could attenuate the bias derived from the uneven distribution of measured covariates, the bias originating from unmeasured ones is unavoidable. Secondly, it is well known that the lactic dehydrogenase level and performance status are important components of lymphoma prognostic factors, but they are not recorded in the SEER database, so we could not include these variables for analysis. Besides, important factors such as whether intestinal lymphoma is germinal center subtype or non‐germinal center subtype,^50^ whether the surgery is urgent or elective,^51^ and whether patients relapse or not are absent in the SEER database, thus limiting the generalizability of our results. In addition, detailed information about chemotherapy and postoperative complications is unavailable in the SEER database, which also limits our further analysis of the effect of chemotherapeutic regimens and complications on prognosis. Nevertheless, the study population was extracted from a national dataset, which could decrease the potential selection bias to some extent. Since both multivariable and PSM analyses were performed, and OS results did not alter significantly, the findings should be valid and stable.

In conclusion, this is the first population‐based real‐world analysis to evaluate the role of surgical treatment in PI‐DLBCL. Our study approves the beneficial effect of surgery on survival outcome in patients with stage I/Ⅱ PI‐DLBCL. For patients who are not suitable for resection, LTD may also be a potential option. The predictable nomogram and its convenient online version could help clinicians evaluate the prognosis and optimize personal guidance for patients with PI‐DLBCL.

## CONFLICT OF INTEREST

The authors declare that there is no conflict of interest.

## AUTHOR CONTRIBUTION

MW and SM designed the study, collected the data, and wrote the paper. WS and YZ contributed to data analysis. SL edited the paper and YH revised the paper. All authors reviewed the paper and approved the final manuscript.

## ETHICAL STATEMENT

The identifiable patient information is not contained in the SEER database, so the approval of an ethics committee was not required.

## Supporting information

Figure S1‐S2

Table S1‐S2

## Data Availability

Data were publicly accessible which can be obtained in the SEER database.
