# Supplementary figures and images for: Surgery shows survival benefit in patients with primary intestinal diffuse large B‐cell lymphoma: A population‐based study
Source: Cancer Med. 2021 May 1;10(10):3474–85. doi: 10.1002/cam4.3882 (PMC8124121; doi:10.1002/cam4.3882)

# Supplemental Figures

**Figure S1**


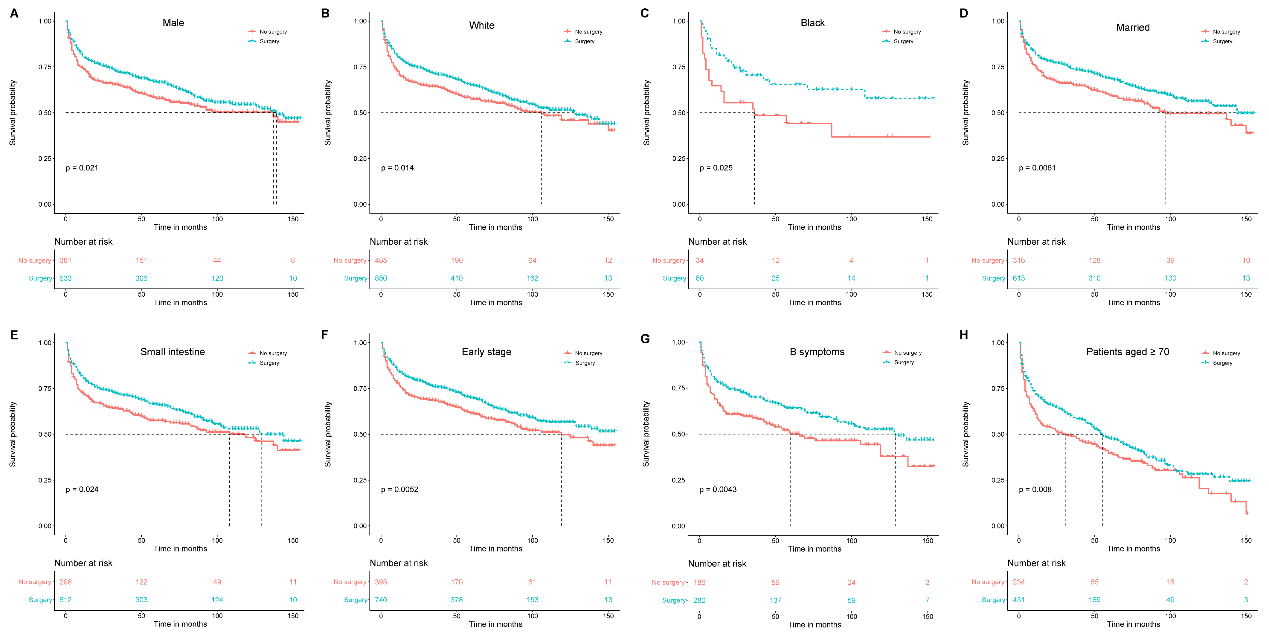


**Figure S2**


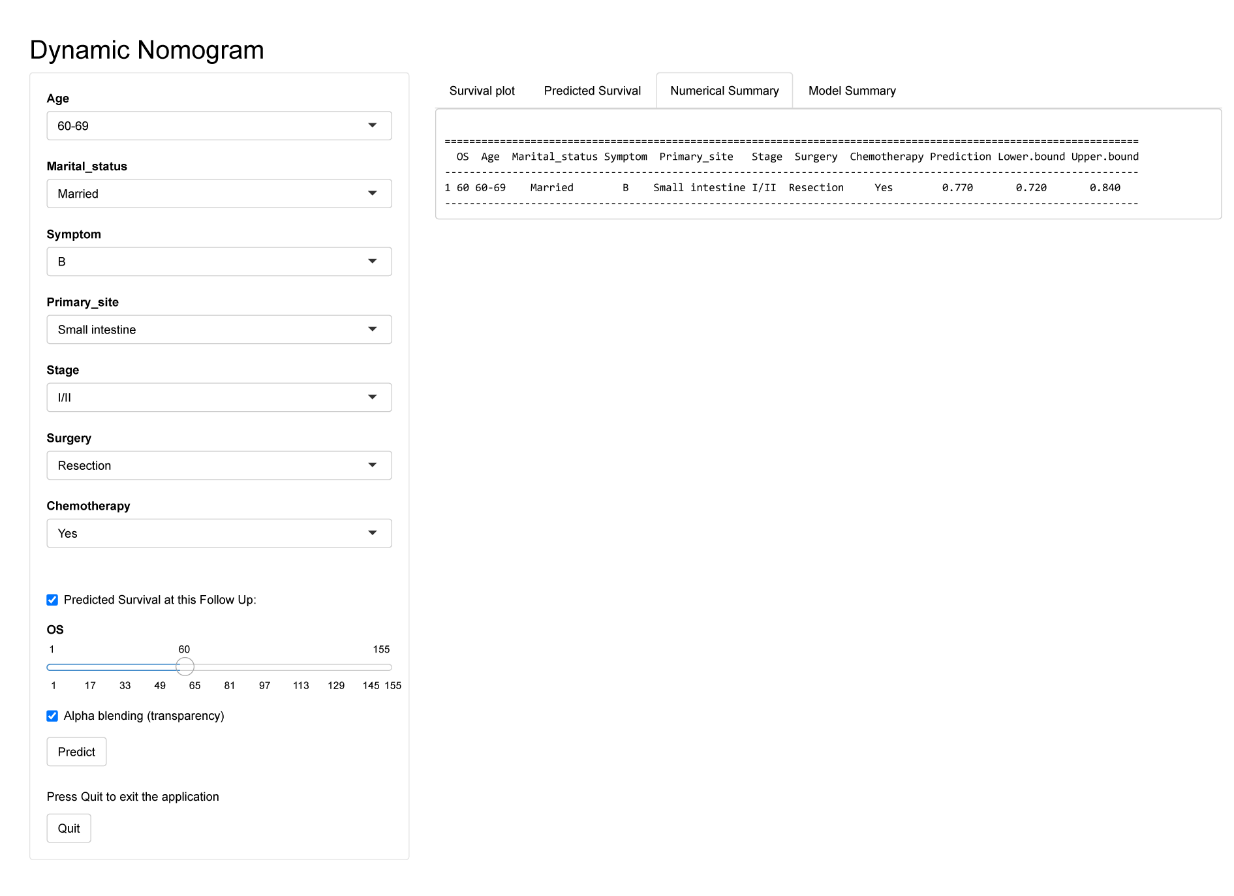

Supplement: Supplementary file 1 — Figure S1‐S2 [file CAM4-10-3474-s001.docx]
