# Supplementary material for: Surgery shows survival benefit in patients with primary intestinal diffuse large B‐cell lymphoma: A population‐based study
Source: Cancer Med. 2021 May 1;10(10):3474–85. doi: 10.1002/cam4.3882 (PMC8124121; doi:10.1002/cam4.3882)
Supplement: Supplementary file 2 — Table S1‐S2 [file CAM4-10-3474-s002.docx]

| Parameters | Before PSM | | | |  | After PSM | | |
| --- | --- | --- | --- | --- | --- | --- | --- | --- |
|  | Total | LTD | Resection | *P** |  | LTD | Resection | *P** |
|  | 695 | 72 (10.4%) | 623 (89.6%) |  |  | 62 | 62 |  |
| Age, years |  |  |  | 0.592 |  |  |  | 0.692 |
| 18-59 | 271 (39.0%) | 33 (45.8%) | 238 (38.2%) |  |  | 27 (43.5%) | 30 (48.4%) |  |
| 60-69 | 189 (27.2%) | 19 (26.4%) | 170 (27.3%) |  |  | 17 (27.5%) | 12 (19.3%) |  |
| 70-79 | 159 (22.9%) | 13 (18.1%) | 146 (23.4%) |  |  | 11 (17.7%) | 14 (22.6%) |  |
| >=80 | 76 (10.9%) | 7 (9.7%) | 69 (11.1%) |  |  | 7 (11.3%) | 6 (9.7%) |  |
| Sex |  |  |  | 0.058 |  |  |  | 0.272 |
| Male | 447 (64.3%) | 39 (54.2%) | 408 (65.5%) |  |  | 34 (54.8%) | 40 (64.5%) |  |
| Female | 248 (35.7%) | 33 (45.8%) | 215 (34.5%) |  |  | 28 (45.2%) | 22 (35.5%) |  |
| Race |  |  |  | 0.848 |  |  |  | 0.734 |
| White | 585 (83.3%) | 59 (82.0%) | 526 (84.4%) |  |  | 52(83.9%) | 55(88.7%) |  |
| Black | 40 (5.9%) | 5 (6.9%) | 35 (5.6%) |  |  | 3 (4.8%) | 2 (3.2%) |  |
| Other | 70 (10.8%) | 8 (11.1%) | 62 (10.0%) |  |  | 7 (11.3%) | 5 (8.1%) |  |
| Marital status |  |  |  | 0.568 |  |  |  | 0.898 |
| Married | 434 (62.4%) | 42 (58.3%) | 392 (62.9%) |  |  | 38 (61.3%) | 39 (62.9%) |  |
| Single | 129 (18.6%) | 13 (18.1%) | 116 (18.6%) |  |  | 10 (16.1%) | 11 (17.7%) |  |
| Other | 132 (19.0%) | 17 (23.6%) | 115 (18.0%) |  |  | 14 (22.6%) | 12 (19.4%) |  |
| Primary site |  |  |  | <0.001 |  |  |  | 0.005 |
| Small intestine | 428 (61.6%) | 44 (61.1%) | 384 (61.6%) |  |  | 43 (69.4%) | 32 (51.6%) |  |
| Colon | 243 (35.0%) | 11 (15.3%) | 232 (37.2%) |  |  | 11 (17.7%) | 27 (43.5%) |  |
| Anorectal | 24 (3.4%) | 17 (23.6%) | 7 (1.1%) |  |  | 8 (12.9%) | 3 (4.9%) |  |
| Stage |  |  |  | 0.141 |  |  |  | 0.839 |
| I/II | 478 (68.8%) | 55 (76.4%) | 423 (67.9%) |  |  | 46 (74.2%) | 45 (72.6%) |  |
| III/IV | 217 (31.2%) | 17 (23.6%) | 200 (32.1%) |  |  | 16 (25.8%) | 17 (27.4%) |  |
| Symptom |  |  |  | 0.110 |  |  |  | 0.828 |
| A | 494 (71.1%) | 57 (79.2%) | 437 (70.1%) |  |  | 49 (79.0%) | 48 (77.4%) |  |
| B | 201 (28.9%) | 15 (20.8%) | 186 (29.9%) |  |  | 13 (21.0%) | 14 (22.6%) |  |

# Supplemental Tables

**Table S1.** Patient characteristics and bias for surgical mode in chemotherapy group.

**P*-value from chi-square tests.

Abbreviation: PSM, propensity score matching; LTD, local tumor destruction.

| Parameters | Before PSM | | | |  | After PSM | | |
| --- | --- | --- | --- | --- | --- | --- | --- | --- |
|  | Total | LTD | Resection | *P** |  | LTD | Resection | *P** |
|  | 320 | 25 (36.6%) | 295 (63.8%) |  |  | 23 | 23 |  |
| Age, years |  |  |  | 0.09 |  |  |  | 0.696 |
| 18-59 | 74 (23.1%) | 8 (32.0%) | 66 (22.4%) |  |  | 7 (30.4%) | 10 (43.5%) |  |
| 60-69 | 50 (15.6%) | 7 (28.0%) | 43 (14.6%) |  |  | 6 (26.1%) | 4 (17.4%) |  |
| 70-79 | 83 (25.9%) | 6 (24.0%) | 77 (26.1%) |  |  | 6 (26.1%) | 4(17.4%) |  |
| >=80 | 113 (35.4%) | 4 (16.0%) | 109 (36.9%) |  |  | 4 (17.4%) | 5 (21.7%) |  |
| Sex |  |  |  | 0.535 |  |  |  | 0.760 |
| Male | 186 (58.1%) | 16 (64.0%) | 170 (57.6%) |  |  | 14 (60.9%) | 15 (65.2%) |  |
| Female | 134 (41.9%) | 9 (36.0%) | 125 (42.4%) |  |  | 9 (39.1%) | 8 (34.8%) |  |
| Race |  |  |  | 0.446 |  |  |  | 0.794 |
| White | 265 (82.8%) | 19 (76.0%) | 246 (83.4%) |  |  | 18 (78.3%) | 16 (69.6%) |  |
| Black | 20 (6.3%) | 3 (12.0%) | 17 (5.8%) |  |  | 2 (8.7%) | 3 (13.0%) |  |
| Other | 35 (10.9%) | 3 (12.0%) | 32 (10.8%) |  |  | 3 (13.0%) | 4 (17.4%) |  |
| Marital status |  |  |  | 0.694 |  |  |  | 0.584 |
| Married | 179 (55.9%) | 16 (64.0%) | 163 (55.3%) |  |  | 15 (65.2%) | 12 (52.2%) |  |
| Single | 50 (15.6%) | 3(12.0%) | 47 (15.9%) |  |  | 2 (8.7%) | 4(17.4%) |  |
| Other | 91 (28.5%) | 6 (24.0%) | 85 (28.8%) |  |  | 6 (26.1%) | 7 (30.4%) |  |
| Primary site |  |  |  | <0.001 |  |  |  | 0.079 |
| Small intestine | 184 (57.5%) | 12 (48.0%) | 172 (58.3%) |  |  | 12 (56.3%) | 9 (56.5%) |  |
| Colon | 128 (40.0%) | 9 (36.0%) | 119 (40.3.%) |  |  | 8 (37.7%) | 14 (38.4%) |  |
| Anorectal | 8 (2.5%) | 4 (16.0%) | 4 (1.4.%) |  |  | 3 (6.0%) | 0 (5.0%) |  |
| Stage |  |  |  | 0.408 |  |  |  | 0.437 |
| I/II | 262 (81.9%) | 22 (88.0%) | 240 (81.4%) |  |  | 20 (87.0%) | 18 (78.3%) |  |
| III/IV | 58 (18.1%) | 3 (12.0%) | 55 (18.6%) |  |  | 3 (13.0%) | 5 (21.7%) |  |
| Symptom |  |  |  | 0.689 |  |  |  | 1.000 |
| A | 241 (75.3%) | 18 (72.0%) | 223 (75.6%) |  |  | 16 (69.6%) | 16 (69.6%) |  |
| B | 79 (24.7%) | 7 (28.0%) | 72 (24.4%) |  |  | 7 (30.4%) | 7 (30.4%) |  |

**Table S2.** Patient characteristics and bias for surgical mode in non-chemotherapy group.

**P*-value from chi-square tests.

Abbreviation: PSM, propensity score matching; LTD, local tumor destruction.
